# 4197 A Landscape Analysis of CTSA websites for Clinical Research Professional Training Opportunities

**DOI:** 10.1017/cts.2020.198

**Published:** 2020-07-29

**Authors:** Karen K Carter, Carolynn Thomas Jones

**Affiliations:** 1 The Ohio State University

## Abstract

OBJECTIVES/GOALS: We conducted a review of CTSA websites to understand the current landscape for CRP institutional professional development and training revealed in the CTSA hub websites. METHODS/STUDY POPULATION: We accessed and reviewed 59 currently funded CTSA hub websites for evidence of CRP training opportunities. Parameters reviewed included: 1) opportunities were specified for CRPs versus K and T trainees; 2) mandated training; 3) leveling; 4) delivery methods/resources; 5) public accessibility; 6) unique features. The website reviews informed a REDCap survey sent to the CTSA Administrators (n = 149) and the Coordinator Taskforce (n = 105) listservs to gain additional knowledge of CRP training available at the institution. A subsequent repeat review of the CTSA hub websites will be conducted to determine evolving trends. RESULTS/ANTICIPATED RESULTS: A total of 40 responded to the survey from 59 CTSA hubs. Survey results are being analyzed. Website review data are being tabulated and the subsequent review of websites will be collected in February. Those findings are pending and will include a comparison of prior findings. 42% of CRP hubs list CRP training within the CTSA hub website. Required onboarding training (beyond CITI certificates) is revealed for some hubs (15%). DISCUSSION/SIGNIFICANCE OF IMPACT: On our initial website review less than half of the CTSA hub websites list specific CRP training on their website. Many were hidden behind firewalls and could not be reviewed for content. The REDCap Survey will provide more granular descriptions of programs. Data from a second website review will be collected for comparison. Based on a preliminary re-review of sites, there is a suggestion of increasing CRP workforce development information. CTSAs are well-positioned to be a central hub for promoting educational excellence of the institutional workforce, for medical centers and in other venues where clinical research is performed.

